# Clinical Pathology Evaluation in Pet Rabbits Vaccinated Against Rabbit Hemorrhagic Disease Virus 2 (RHDV2)

**DOI:** 10.3390/ani14203029

**Published:** 2024-10-19

**Authors:** Chris Griffin, Salina Locke, Fabiano Montiani-Ferreira, Andressa Lopes Grego, Jeny Soto, Carolyn Cray

**Affiliations:** 1 Griffin Avian & Exotic Veterinary Hospital, Kannapolis, NC 28083, USA; 2 Avian and Exotic Animal Care, Raleigh, NC 27617, USA; 3 Departamento de Medicina Veterinária, Universidade Federal do Paraná, Curitiba 80035-050, PR, Brazil; 4 Division of Comparative Pathology, Department of Pathology & Laboratory Medicine, University of Miami Miller School of Medicine, Miami, FL 33136, USA

**Keywords:** antibody, biochemistry, ELISA, hematology, lagomorph, RHDV2

## Abstract

A recombinant vaccine for rabbit hemorrhagic disease virus 2, a highly pathogenic virus, was granted emergency use authorization in the United States after the detection and spread of the virus starting in 2018. The goal of the current study was to assess pet rabbits (n = 29) through physical examination and routine clinical pathology testing using repeated assessments post-vaccination. In addition, seroconversion was also monitored after the initial vaccination and booster vaccination. Neither owners nor clinicians detected any physical abnormalities in relationship to the vaccine protocol. Hematological and clinical biochemistry testing showed some changes although median values were within species specific reference intervals. A significant increase in antibody levels was observed at day 21 (post-initial vaccination) and day 49 (post-booster vaccination) versus that present at baseline (*p* < 0.0001). However, variability in study rabbits was noted with some individuals showing low antibody levels as well as a lower overall response in older rabbits (r = −0.56, *p* = 0.006). A second cohort of rabbits was assessed at 11–12 months post-initial vaccination. In this second group, antibody levels were not significantly different from baseline levels (*p* = 0.21). Additional studies should be conducted to further define the variability in seroconversion and the term of protection in pet rabbits as the industry moves forward in the optimization of RHDV2 vaccines.

## Simple Summary

Rabbit hemorrhagic disease virus 2 (RHDV2) was reported in the United States in 2018 and has since spread across most of the country where it has a potential for adverse impacts on ecosystems, economic losses, and pet rabbit health. Using an emergency use authorized vaccine, seroconversion and protection was demonstrated in 3- to 7-week-old specific pathogen free laboratory rabbits. The purpose of the current study was to examine this protocol in pet rabbits, which would be inclusive of varied breeds and ages. Importantly, no significant differences were observed in routine clinical pathology measures. Seroconversion was also observed but was variable, and lower levels of seroconversion were associated with increased age. In general, while variable outcomes to vaccination are expected, these preliminary data underlie the importance of examining the response of pet rabbits to help optimize new vaccine protocols. We propose this will complement traditional vaccine studies, aid in maintaining the health of pet rabbits, and help in the ultimate control of this highly infectious virus.

## 1. Introduction

Rabbit hemorrhagic disease virus (RHDV) is a calicivirus, which was first described in conjunction with a high mortality event in China in 1984 [1,2]. It gained essentially a worldwide distribution in the following years and, in 2010, a second serotype known as RHDV2 was identified in Europe [3,4]. By 2018, the virus was identified in the United States and mortality events have been documented since that time as the virus spread across the country [3]. RHDV has long been recognized to be economically important to rabbit fur and meat industries. Notably, RHDV2 has been shown to have a wider host range than RHDV infecting both domestic and wild rabbits and hares [3]. Exotic animal veterinarians and pet rabbit owners have become aware of the potential for RHDV2 infection, which led to an increased interest in rabbit health and welfare at a time of increased popularity in ownership of rabbits as companion animals. As with farmed rabbits, pet rabbits can be subject to many different stressors related to husbandry and poor sources of enrichment [5,6].

Multiple vaccines have been produced for RHDV, including using attenuated and inactivated forms of the virus [4]. In addition, recombinant vaccines have been developed and studied for both RHDV and RHDV2 [7,8]. This latter approach was also utilized in the United States where emergency use authorization (EUA) was granted to a novel baculovirus derived recombinant vaccine produced by Medgene Labs (Brookings, SD, USA). This virus expresses VP60, which is the main target of immune responses to RHDV and RHDV2 viruses [9,10]. The initial study of 22 laboratory rabbits (n = 9 vaccinated, n = 13 control) demonstrated protection after experimental infection followed by a second study describing seroconversion and immunity [9,11].

The two studies using the EUA vaccine involved the use of 3- to 7-week-old specific and opportunistic pathogen free New Zealand White laboratory rabbits and examined seroconversion and protection with viral challenge [9,11]. As an important contrast, the present study was conducted using pet rabbits of various breeds and ages that received the EUA vaccine as part of a recommended health program from their veterinarian. The primary goals were two-fold. First, to assess changes in physical condition and document any changes in routine hematological or clinical chemistry measures. Second, to examine seroconversion for the VP60 antibody via ELISA after initial vaccination and booster vaccine application. In addition, rabbits were assessed for antibody levels to *Encephalitozoon cuniculi* during the study after receipt of anecdotal reports of reactivation of *E. cuniculi* disease post-vaccination.

## 2. Materials and Methods

### 2.1. Rabbits

Samples from patients from two clinics in North Carolina were used in this study. Rabbits were assessed with the permission of the owner after injection with the RHDV2 vaccine (Medgene, Brookings, SD, USA). On entry of their pets into the study, owners were asked to notify the clinic of any abnormalities observed during and after the vaccination protocol including but not limited to inappetence and lethargy. Prior to vaccination and booster vaccination, the veterinary staff also queried the owners and conducted a general physical examination. The latter was inclusive of a visual examination of the eyes, ear, nose, mouth, and coat; stethoscope examination of the heart, lungs, and gastrointestinal system; palpitation of the body; intraoral examination, and ear exams. Body condition (BCS) was scored as follows: 1–5 scale (with 3 as normal, 5 as very obese, and 1 as emaciated). Patient nutrition was also evaluated by survey and discussion of the owner and included a balanced variety of hay, pellets, and fresh foods.

A total of 29 patients participated in the study with the median age of 2 years (CI: 1–3) including 12 female and 17 male animals in good health representing 11 different breeds. The median BCS was 3.0 (CI: 2–4). Animals were examined and had blood samples taken on day 0 or 1, 7, 21, and 49 (Table 1). Seven patients had blood samples taken on day 1 rather than day 0 and were examined again on day 7. Fifteen patients had evaluations at the start, day 21, and day 49. In total, 13 of the 15 rabbits were also examined on day 7. Due to lack of owner compliance, the final 7 patients had incomplete timepoints, and data from these animals were not used in the statistical analyses. A second cohort of samples were from rabbits that had received the vaccine and booster 330–365 days previously. The median age was 3.5 years (CI: 2.3–4.9) including three female and eight male animals in good health.

### 2.2. Samples

Samples were collected without sedation from the lateral saphenous vein while being gently restrained. Samples included whole blood in EDTA tubes, freshly made blood tubes, and heparinized plasma. Samples were shipped on cold pack using an express shipper to the laboratory.

### 2.3. Clinical Pathology Testing

Complete blood count was performed using a Genesis hematology analyzer (Oxford Science, Inc., Oxford, CT, USA) with a manual differential of Wright Giemsa-stained blood smears and packed cell volume. Routine biochemistry testing was conducted using a Vitros 5600 chemistry analyzer (Ortho, Rochester, NY, USA). Testing included glucose, BUN, creatinine, calcium, phosphorus, total protein, and ALT. C-reactive protein (CRP) was quantitated using a rabbit specific reagent and VetBio1 analyzer (Veterinary Biomarkers, West Chester, PA, USA). All analyzers were maintained according to manufacturer instructions.

### 2.4. Serological Testing

Study samples were analyzed using a commercially available ELISA kit coated with a recombinant RHDV VP60 polypeptide (MyBiosource, San Diego, CA, USA). The ELISA was used per manufacturer instructions and included a positive viral antigen and negative control antigen well configuration. Anti-rabbit IgG was obtained from Southern Biotech (Birmingham, AL, USA). Results are expressed as optical density units. Twenty samples were also tested by a recently described RHDV2 ELISA [9]. These samples were inclusive of day 0, 21, 49 timepoints for six rabbits and day 0 and 49 timepoints for one rabbit. A strong positive correlation was observed between the results of the two assays (r = 0.80, *p* < 0.0001). As sample volume permitted, additional testing was performed to examine samples for antibody to *Encephalitozoon cuniculi* using the methodology previously described [12].

### 2.5. Statistics

All data were examined for normality using the D’Agostino–Pearson test. Non-normally distributed data from the repeated measures were tested using the Friedman test (to analyze how titers have changed at the different evaluation times in the studied population) and the Kruskal–Wallis test (for comparing independent subgroups at specific blood collection at baseline and days 21 and 49). Data with normal distribution were tested using repeated measures ANOVA. The Mann–Whitney test was used to compare the day 330–365 serological results to baseline. Spearman’s correlation analyses were used for comparison of assay data and patient morphometrics. *p* values < 0.05 were considered significant. Sample size analysis for paired samples indicated a minimum required number of paired observations of five, considering an α-level of 0.05, β-level of 0.20 (power of 80%), a mean difference of 1.4 and a standard deviation of differences of 0.76 (1.96 times the initial standard deviation). All statistics were performed using MedCalc software (MedCalc Software Ltd., Ostend, Belgium; version 22.014)

## 3. Results

Throughout the study, the owners did not report any clinical signs in their pet rabbits such as pain, swelling, inappetence, or any behavioral changes. Owners were able to contact the participating clinics with any concerns about their pets and were otherwise prompted for this information during appointments for blood testing and booster vaccination. No clinical abnormalities were observed on the physical exam.

Based on repeated measures statistical analyses, there were no significant differences in any hematological parameters, biochemistry analytes, or CRP levels (Table 2). There was a mild increase in levels of BUN by day 21 and through day 49, although all values were within reference intervals for this species, and there were no significant differences (*p* = 0.06). The observed change in BUN was not associated with age, sex, or breed.

Seven rabbits were examined at day 1 following the initial vaccination as well as at day 7. Hematological and biochemistry results were within reference intervals on day 1. One rabbit showed a mild increase in CRP on day 7. An additional thirteen rabbits were examined on days 0 and 7 (Table 1). Of this group, four rabbits showed elevated CRP levels on day 7 but two of the four animals had increased levels present on day 0.

**Figure 1:**
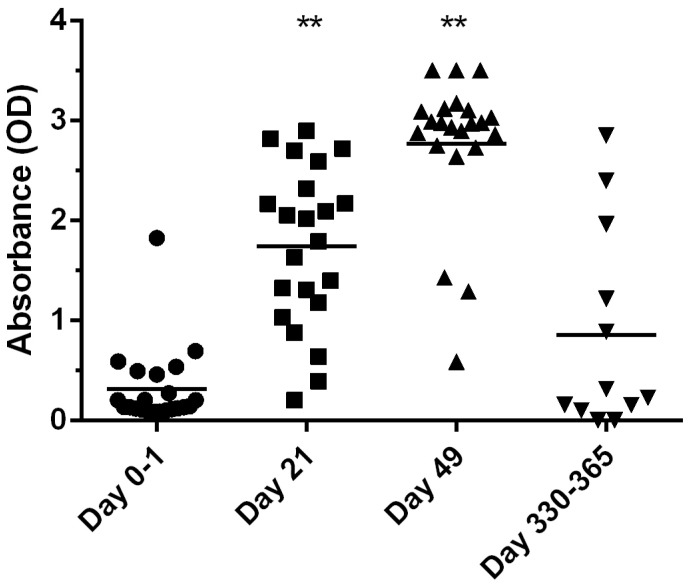
Antibody levels to RHDV at baseline (day 0–1, n = 22), day 21 (n = 22), and day 49 (n = 22) in the original cohort of samples. Additional samples were collected from a second cohort of rabbits on day 330–365 (n = 11). Statistical difference of *p* < 0.0001 versus day 0–1 indicated by **.

Serological data are presented in Figure 1. A significant increase in antibody levels was observed between day 0 and day 21/day 49 (*p* < 0.0001). In addition, there was a significant increase in levels between day 21 and day 49 (*p* < 0.0001). There was no significant difference between day 0 and day 330–365 (*p* = 0.22).

**Figure 2:**
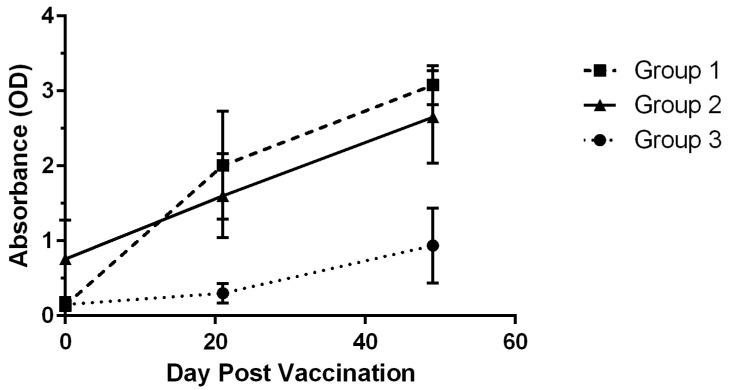
Antibody levels over time as observed in three subgroups of rabbits. Mean and standard deviation (error bars) are represented. Data are inclusive of n = 22 rabbits with complete protocol timepoints.

Based on antibody levels, three groups of rabbits were defined (Figure 2). Group 1, 14/22 rabbits, showed a significant increase in antibody levels on day 21 and a further increase by day 49. Group 2, 6/22 rabbits, showed elevated levels at the time of baseline assessment; this group was significantly different from the other groups at the baseline assessment (*p* = 0.002). Group 3, 2/22 rabbits showed a low response on day 21 and were significantly different from both other groups (*p* = 0.02). By day 49, group 3 was different from group 1 (*p* = 0.008). When the day 49 data were examined by breed, there was no significant difference observed (*p* = 0.21). No differences were observed when comparing levels of male vs. female rabbits (*p* = 0.24) nor castrated vs. ovariohysterectomized rabbits (*p* = 0.29). On comparison of castrated/ovariohysterectomized rabbits versus non castrated/ovariohysterectomized rabbits, a significant difference was observed with a mild increase in median antibody levels in the latter group (*p* = 0.04). A significant negative correlation was observed with antibody levels with increased age (r = −0.56, *p* = 0.006).

Of the total study rabbits, 18/29 were seronegative for *E. cuniculi* and no rabbit seroconverted during the study. Of the 11 rabbits which were seropositive at the start of the study, only one rabbit showed a 2-fold increase in antibody titer by day 49. This rabbit remained clinically normal.

## 4. Discussion

In this study, although a comprehensive toxicological review was not conducted post-vaccination, the pet owners nor the participating clinicians noted no adverse side effects. This finding was consistent with reports of other recombinant vaccines against RHDV [10]. Moreover, changes in hematology parameters were not observed. This is an important observation as clinical pathology testing is commonly performed in vaccine toxicological studies [13]. The absence of hematological changes may be related to the time periods which were examined post-vaccination (day 21, day 49) but a subset of rabbits was examined at day 1 and day 7 and no significant differences were present. Clinical chemistry parameters also showed no significant differences. The origin of a mild increase in BUN is not known. Importantly, the values were within the reference intervals and the rabbits remained normal throughout the vaccine protocol. The change in BUN may be related to the immune response to the antigen as well as the adjuvant. Aluminum hydroxide adjuvants, common to many vaccines including the Medgene RHDV2 product, have been shown to concentrate in tissues of rabbits including the kidney at least through 28 days post-intramuscular injection [14,15]. As the rabbits remained clinically normal throughout and after the study period, no additional studies were undertaken to investigate possible renal changes in the pet rabbits in this study.

Levels of CRP were evaluated, as this is a known major acute phase protein in the rabbit, and this biomarker has been proposed to be an accurate indicator of inflammation for vaccine safety studies in this species [12,16]. Notably, no significant differences were observed in the current study. In a study of various adjuvants, CRP levels were observed to peak at day 1 and continue to be present through day 7 [16]. In the current study, seven rabbits were examined on day 1 and all were within reference intervals. On day 7, four rabbits were found to have elevated CRP although two out of four rabbits had elevated CRP at day 0. By day 49, CRP had normalized in the majority of rabbits. These changes may reflect a variability in the response to vaccination by the study animals or possible subclinical inflammation. Notably, the rabbits were normal during the course of the study and for a minimum of 3 months after the completion of the study.

In the present study, a commercially available ELISA for the detection of antibody to RHDV was utilized given the absence of such a product in the U.S. for the detection of RHDV2 antibody. This ELISA utilizes a recombinant VP60 antigen, which also serves as the target for the RHDV2 vaccine, and this type of indirect ELISA has previously been reported to be sensitive for the detection of antibodies post RHDV vaccination while others have noted there is not 100% serological agreement as reflected in the differing level of reactivity found with the two antigens [8,10,17]. Of note, as more than 90% homology has been reported between the VP60 found in RHDV and various strains of RHDV2, this would be presumed to be a suitable ELISA for the detection of antibody to the latter [10]. While a comprehensive validation of the present ELISA was not conducted, it is notable that the RHDV ELISA was strongly correlated with a recently described laboratory developed RHDV2 ELISA [9].

Notably, significant increases in antibody levels were observed 21 days after the first vaccination and then again 4 weeks (day 49) after the booster. These findings are consistent with those reported by other recombinant vaccines for RHDV and RHDV2 [7,8,10,18,19,20]. Interestingly, three cohorts of rabbits could be defined. In 63.6% of rabbits, the most common response was observed in group 1 with significant increases in levels occurring on day 21 and again after receiving the booster. In group 2, 27.3% of rabbits displayed higher levels at the baseline assessment and levels continued to increase after vaccination to a level comparable to group 1. The higher baseline levels may reflect possible exposure to RHDV2. In this group, two rabbits had unknown backgrounds as they were acquired through adoption and one rabbit had outdoor access. Other RHDV serological studies have also observed antibody levels in the absence of vaccination and have proposed this is reflective of contact with other nonpathogenic caliciviruses [21]. Group 3, with 9.1% of rabbits, represented a putative low responder group with low levels at day 21. By day 49, levels increased in the rabbits available for reassessment but continued to lag the response of Group 1 rabbits. In this group, one animal had an elevated CRP level at the time of the first vaccination, suggestive of a subclinical inflammatory process. Another animal with low antibody levels on day 21, and which did not continue in the study, was later diagnosed with thymoma. Overall, variability in responsiveness is an expectation to vaccination [22]. In a recent publication examining immunity generated by the EUA RHDV2 vaccine, it was noted that young specific and opportunistic pathogen-free New Zealand White rabbits had a vigorous response ranging from titers of 1:1600 to 1:6400, while rabbits examined over 200 days later had titers ranging from 1:400 to 1:6400 [9]. Notably, in a report using a different RHDV2 vaccine, fatal infection with the virus was observed in small cohort of pet rabbits that had been vaccinated perhaps reflective of the inconsistency of response and/or the efficacy of the vaccine [23]. These reports and the preliminary data from the present study underlie the importance of also examining vaccines in pet rabbits.

While all rabbits receiving the vaccine protocol were considered clinically healthy by the initial physical examination, there should be an expectation in differences based on immunogenetics, age, subclinical disease, and other unknown stressors. In the present study, there was no association between any particular breeds and seroconversion, albeit the number of individuals from the same breed was likely not enough to perform such an analysis. Interestingly, non-castrated/ovariohysterectomized rabbits were observed to have a slightly higher yet significant increase in antibody levels by day 49. A significant difference was also found with lower overall levels on day 49 in older animals. These observations may be concurrent as with increased age, animals are more likely to be castrated and ovariohysterectomized. In a study of RHDV antibody in clinically normal farmed rabbits, both a higher seroconversion was observed in younger and female rabbits [24]. Female rabbits were also observed to make more antibody in response to immunization with experimental antigens [25]. In mice, dogs, and humans, antibody production has been found to vary with sex and age, with the hypothesis that this is related to changing levels of hormones [26,27,28]. In total, these observations are interesting considering the preliminary findings of the current study. Importantly, seroconversion was observed regardless of age or castration/ovariohysterectomy status, but further studies should be conducted to understand the influence of these factors when optimizing vaccine protocols in rabbits.

Neutralizing antibodies have been shown to protect from severe disease and death from RHDV, and higher titers have been associated with protection [2,4,7]. Levels of neutralizing antibody were not addressed in the current study nor were antibody levels fully titrated. As CD8+ T cytotoxic cells are also important effector cells during viral infection and, importantly, one cannot equate protection with either response exclusively [7]. However, to provide some comparison of antibody levels post vaccination, a second cohort of samples from rabbits that had received the vaccine and booster 11–12 months previously were assessed. As described for the current EUA vaccine and other RHDV vaccines, the levels waned in many of the rabbits and was not statistically different from baseline values [7,9]. The current recommendation is a booster application after one year, and this recommendation was supported by the increased titer observed using this protocol in a small treatment group by the manufacturer [9]. A future study inclusive of a larger sample set should be conducted on pet rabbits.

Lastly, anecdotal reports post-vaccine had indicated reactivation of *E. cuniculi* infection in some rabbits (C. Cray, personal communication). As *E. cuniculi* is a common latent infection in rabbits that is known to be affected by increased stressors, this association could be expected. In the present study, all seropositive rabbits remained clinically normal, including one rabbit that previously had suspected infection.

This study was designed to obtain preliminary data on the use of the emergency use authorized vaccine for RHDV2 in pet rabbits. It is limited in sample size and diversity of breed and age. In addition, the assessment of pet health post-vaccination by the owner may have been variable although the collaboration with two exotic animal hospitals that utilized similar protocols in physical examination, body scores, and sampling techniques was an advantage. Lastly, rabbits were not followed through a complete year to assess, through paired measures, the waning antibody levels prior to the one-year booster vaccination. Future studies should consider these possible variables and include a cohort of rabbits with chronic disease to more fully assess the efficacy of the new vaccine.

## 5. Conclusions

The novel vaccine for RHDV2 has demonstrated protective efficacy in a small number of very young specific pathogen-free New Zealand White rabbits [9,11]. In the current study with varied breeds, age, and sex, no clinically significant changes in physical condition, hematology, or biochemistry were observed. However, differences were apparent in serological response to the vaccine protocol. This may reflect a difference in the immunogenetics of the various rabbit breeds, age-related differences, or the presence of possible subclinical disease that may have influenced seroconversion [29]. Additional studies should be conducted to understand this possible failure rate as the industry moves forward in the optimization of RHDV2 vaccines.

## Figures and Tables

**Table 1:** Study animal information—animals with all protocol timepoints only.

Descriptor	Information
Breed	Holland Lop = 10, Lionhead = 4, Lop Mix = 2, Mini Lop = 1, New Zealand White = 1, American = 1, American Mix = 1, Polish = 1, Flemish Giant = 1
Age	median age of 2 years (CI: 1–4)
Sex	11 castrated, 5 ovariohysterectomized, 3 male, 3 female
Day 1 and 7, 21, 49 sampling	7 patients—Lionhead = 3, Holland lop = 2, Flemish Giant = 1, New Zealand White = 1; castrated = 4, ovariohysterectomized =1, male = 1, female = 1; median age 1 year (CI 1.0–3.0)
Day 0, 21, 49 sampling	15 patients—Holland Lop= 8, Lop mix = 2, Lionhead = 1, American =1, Mini Lop = 1, American Mix = 1, Polish = 1; castrated = 7, ovariohysterectomized = 4, male = 2, female = 2; median age 3 years (CI: 1–4); note: 13/15 rabbits also examined on day 7

**Table 2:** Hematology and biochemistry measurands pre- and post-vaccination. Data are inclusive of 15 rabbits (except as noted) that were assessed on days 0, 21, 49. Median and 25th/75th percentiles are presented. Reference intervals were established within the laboratory.

Measurand	Day 0	Day 21	Day 49	*p*-Value	Reference Interval
WBC × 10^3^/µL	6.4 (5.2–9.7)	5.9 (4.9–7.1)	5.5 (4.9–6.7)	0.27	4.3–12.9
RBC × 10^6^/µL ^a^	6.09 (5.39–7.22)	6.46 (5.84–6.71)	5.99 (5.76–6.74)	0.86	4.74–7.31
Hemoglobin, g/dL ^a^	11.5 (10.4–13.2)	12.0 (11.0–12.8)	11.6 (11.3–12.6)	0.92	9.4–15.9
Hematocrit, % ^a^	40 (37–49)	42 (37–45)	44 (40–49)	0.48	34–45
Segmented neutrophils × 10^3^/µL	2.4 (1.5–3.1)	2.1 (1.9–3.7)	2.1 (1.4–3.2)	0.99	1.1–3.2
Band neutrophils × 10^3^/µL	0 (0–0)	0 (0–0.04)	0 (0–0)	0.74	0–1
Lymphocytes × 10^3^/µL	3.0 (2.2–5.3)	3.0 (2.2–4.6)	3.3 (1.9–3.9)	0.17	1.6–8.8
Monocytes × 10^3^/µL	0.47 (0.11–0.57)	0.27 (0.10–0.46)	0.14 (0.06–0.25)	0.75	0–0.5
Eosinophils × 10^3^/µL	0.06 (0–0.18)	0 (0–0.06)	0 (0–0.07)	0.15	0–0.4
Basophils × 10^3^/µL	0.07 (0–0.44)	0.2 (0–0.36)	0.1 (0.06–0.31)	0.87	0–0.3
Platelets × 10^3^/µL ^a^	270 (200–364)	285 (243–339)	233 (126–312)	0.40	162–571
Glucose, mg/dL ^a^	133 (118–154)	155 (133–161)	131 (121–149)	0.19	81–145
Total Protein, g/dL ^b^	7.0 (6.6–7.6)	7.4 (6.9–7.7)	6.9 (6.5–7.4)	0.23	5.4–7.7
BUN, mg/dL ^a^	20.0 (15.0–23.0)	20.0 (18.0–23.0)	22.0 (16.0–26.5)	0.06	11–24
Creatinine, mg/dL ^a^	1.0 (0.8–1.1)	1.0 (0.8–1.1)	1.0 (0.8–1.1)	0.96	0.6–1.0
Calcium, mg/dL ^a^	14.0 (13.8–14.9)	13.9 (13.7–15.4)	13.6 (12.9–14.2)	0.36	9.5–14.0
Phosphorus, mg/dL ^c^	3.2 (2.7–3.9)	3.6 (2.9–4.1)	3.4 (2.9–4.5)	0.66	3.2–6.0
ALT, U/L ^a^	52 (47–89)	53 (43–81)	62 (40–97)	0.92	27–108
CRP, mg/L ^a^	7.6 (5.1–25.1)	11.1 (5.8–17.2)	5.5 (5.0–12.5)	0.25	0–11.9

^a^ n = 13; ^b^ n = 14; ^c^ n = 11.

## Data Availability

The raw data will be made available by the authors upon request.
